# Direct Mechanocatalysis: Palladium as Milling Media and Catalyst in the Mechanochemical Suzuki Polymerization

**DOI:** 10.1002/anie.201911356

**Published:** 2019-11-07

**Authors:** Christian G. Vogt, Sven Grätz, Stipe Lukin, Ivan Halasz, Martin Etter, Jack D. Evans, Lars Borchardt

**Affiliations:** ^1^ Inorganic Chemistry I Technische Universität Dresden Bergstrasse 66 01062 Dresden Germany; ^2^ Inorganic Chemistry I Ruhr-Universität Bochum Universitätsstrasse 150 44780 Bochum Germany; ^3^ Laboratory for Green Synthesis Ruđer Bošković Institute Bijenicka 54 HR-10000 Zagreb Croatia; ^4^ Deutsches Elektronen-Synchrotron (DESY) 22607 Hamburg Germany

**Keywords:** heterogeneous catalysis, mechanochemistry, poly(*para*-phenylene), sustainable chemistry, Suzuki cross-coupling

## Abstract

The milling ball is the catalyst. We introduce a palladium‐catalyzed reaction inside a ball mill, which makes catalyst powders, ligands, and solvents obsolete. We present a facile and highly sustainable synthesis concept for palladium‐catalyzed C−C coupling reactions, exemplarily showcased for the Suzuki polymerization of 4‐bromo or 4‐iodophenylboronic acid giving poly(*para*‐phenylene). Surprisingly, we observe one of the highest degrees of polymerization (199) reported so far.

## Graphical abstract

**The milling ball is the catalyst**: In a mechanochemical Suzuki polymerization of 4‐bromo‐ or 4‐iodophenylboronic acid yielding poly(*para*‐phenylene) the classical Pd catalysts could be replaced by solid Pd milling balls, enabling outstanding degrees of polymerization.

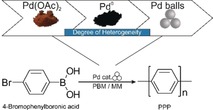

The formation of C−C bonds is an important tool in organic^1^, ^2^ and polymer chemistry.^3^, ^4^, ^5^ There are many cross‐coupling reactions established to create those bonds such as Negishi,^6^, ^7^ Mizoroki–Heck,^8^, ^9^ Sonogashira,^10^ and Suzuki–Miyaura^5^, ^11^ coupling. In all these, palladium species are required as a catalyst, mainly brought in as complexes such as tetrakis(triphenylphosphine)palladium(0).^1^ In most cases, the catalyzed reactions are run homogeneously, although there are discussions and studies in the literature about heterogeneous analogues.^12^, ^13^, ^14^ In the recent past, several of the aforementioned reactions have been performed in a solvent‐free manner in ball mills.^15^, ^16^, ^17^, ^18^, ^19^ These mechanochemical reactions proved advantageous, being extremely swift and consuming less energy while the solvent‐free reaction environment significantly reduces the amount of waste produced and circumvents any possible solubility issues.^20^, ^21^ However, often the catalyst salts used in these solvent‐free protocols are still the same as those in conventional solution‐based processes.

Utilizing this pathway, we have recently demonstrated that a mechanochemical Suzuki polymerization is possible even with simple palladium(II) acetate catalyst.^22^ This solid‐state approach yielded poly(*para*‐phenylene) (PPP) much faster than by solution or electrochemical synthesis. In addition, the reached degree of polymerization (DP) was greatly elevated.^22^ These materials are promising conducting polymers in opto‐ and microelectronics.^23^, ^24^

In this contribution, we advance the system of a mechanochemical Suzuki polymerization considerably by using the milling equipment itself as the catalyst. This represents a considerable advance since processing steps are reduced and separation is greatly simplified. This concept which we will call “direct mechanocatalysis” was motivated by pioneering work of the Mack group, who replaced copper(I) iodide in a Sonogashira coupling by using a milling vessel made of copper and copper balls.^25^ They also developed a rapid and efficient azide–alkyne‐type click reaction that does not require any copper(I) salt in the reaction mixture.^26^ Moreover, they also extended the range of possible metals by using nickel pellets as milling balls, which proved to be an excellent catalyst in the cycloaddition of alkynes yielding cyclooctatetraenes.^27^ Other groups picked up the methodology and showed that even stainless steel milling equipment could serve as a catalyst for hydrogen generation^28^ and the reduction of organic compounds.^29^ Herein, we demonstrate that milling balls made out of palladium metal (one of the most frequently used transition metal catalysts^30^) catalyze the Suzuki cross‐coupling reaction. No additional catalyst powder, no ligands, and no solvent has to be applied.

With this goal in mind, we proceeded stepwise by adapting the well‐known Pd(OAc)_2_‐catalyzed Suzuki polymerization of 4‐bromophenylboronic acid yielding poly(*para*‐phenylene), by first applying Pd^0^ black metal powder, and finally solid Pd milling balls (Scheme 1). In detail, we looked at how the milling material, milling time, catalyst concentration, halide function of the monomer, and mill type influenced the reaction yield and DP.

**Scheme 1:**
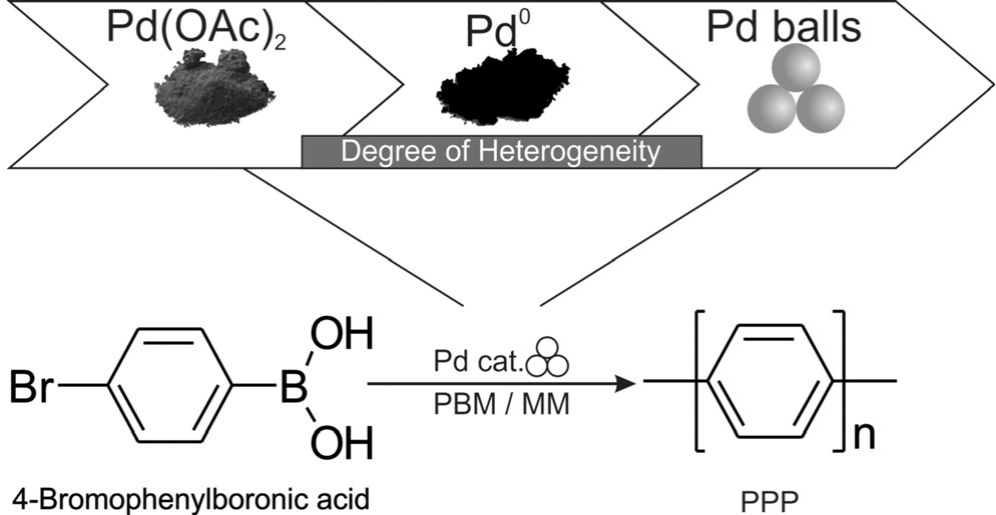
Mechanochemical Suzuki polymerization reaction of 4‐bromophenylboronic acid to give poly(*para*‐phenylene) (PPP). The Pd catalyst was subsequently advanced and simplified from a Pd salt to Pd black and finally to simply Pd milling balls.

In our standard synthesis we used 1.454 g (7.2 mmol) 4‐bromophenylboronic acid, 8.510 g (61 mmol) K_2_CO_3_ as a base, and 4.7 mol % (0.34 mmol) catalyst in a planetary ball mill (PBM) with 22 ZrO_2_ milling balls (10 mm diameter, 3.19 g each) placed in a 45 mL ZrO_2_ milling vessel. The milling time ranged from 2 to 6 hours (see Table 1). The resulting reaction mixture was washed and dried overnight (for characterization see Section 2 in the Supporting Information).

**Table 1:** Poly(*para*‐phenylene) obtained by mechanochemical Suzuki polymerization in a planetary ball mill with Pd(OAc)_2_ and Pd black as catalysts.

	Sample code	Vessel/ ball material	Reaction time [h]	Amount of catalyst	DP	Yield of insoluble polymer [%]^[a]^	Halide function of monomer
Reference PBM	PPP_PBM_‐Ref^22^	ZrO_2_	0.5	9.3 mol % Pd(OAc)_2_	69	47	Br
Pd black/PBM	PPP_PBM_‐1	ZrO_2_	4	4.7 mol % Pd black	18	59	Br
	PPP_PBM_‐2	steel	4	4.7 mol % Pd black	13	56	Br
	PPP_PBM_‐3	WC	4	4.7 mol % Pd black	10	81^[b]^	Br
	PPP_PBM_‐4	Si_3_N_4_	2	4.7 mol % Pd black	34	19	Br
	PPP_PBM_‐5	Si_3_N_4_	3	4.7 mol % Pd black	45	14	Br
	PPP_PBM_‐6	Si_3_N_4_	4	4.7 mol % Pd black	56	22	Br
	PPP_PBM_‐7	Si_3_N_4_	5	4.7 mol % Pd black	40	19	Br
	PPP_PBM_‐8	Si_3_N_4_	6	4.7 mol % Pd black	32	34	Br
	PPP_PBM_‐9	Si_3_N_4_	4	2.3 mol % Pd black	55	20	Br
	PPP_PBM_‐10	Si_3_N_4_	4	0.5 mol % Pd black	48	5	Br
	PPP_PBM_‐11	Si_3_N_4_	4	4.7 mol % Pd black	0	0	Cl
	PPP_PBM_‐12	Si_3_N_4_	4	4.7 mol % Pd black	79	66	I

DP and yield measured five times with a standard deviation of ±5 % [a] Yield calculated from the mass of insoluble polymer after washing with water, 10 wt % HCl, ethanol, and acetone. In the organic washing solutions, smaller oligomers could be detected (see Section 1.2 in the Supporting Information). [b] Overestimated due to impurities from WC abrasion. Densities of the milling materials and weight of the milling balls: ZrO_2_ 5.7 g cm^−3^, 3.19±0.05 g, steel 7.7 g cm^−3^, 4.02±0.02 g, WC 14.3 g cm^−3^, 7.20±0.26 g, Si_3_N_4_ 3.25 g cm^−3^, 1.94±0.03 g.

First, we conducted the reference reaction using Pd(OAc)_2_ with milling balls and vessel made from ZrO_2_ and obtained PPP_PBM_‐Ref in 47 % yield and with a DP of 69 after 30 min milling time.^22^ After that we switched to Pd^0^ black metal powder as the catalyst. The resulting material (in the following exemplarily shown for PPP_PBM_‐6) was crystalline (see powder X‐ray diffraction (PXRD) pattern in Figure 1 A),^48^ the C/H ratio obtained by elemental analysis is close to the theoretical value (see Table S2), and the material showed the characteristic Fourier transform infrared (FTIR) spectrum of PPP (Figure 1 B and Figure S2). FTIR spectroscopy was used to determine the degree of polymerization (DP) by measuring the ratio of the band at 690 cm^−1^ (attributed to terminal phenyl rings) to that at 805 cm^−1^ (principal band of *para‐*substituted benzene rings)^31^, ^32^ (for further information see Section 1.1 in the Supporting Information). The materials were also investigated via Raman spectroscopy (Figure 1 C and Figure S3) where characteristic bands were observed at 1220 cm^−1^ (ν_intra‐ring_(C−C)), 1280 cm^−1^ (ν_inter‐ring_(C−C)), and 1600 cm^−1^ (δ_in‐plane_(C−H)).^33^ Fitting the spectral peaks by a Lorentzian function^34^ and comparing the intensities of the peaks at 1220 cm^−1^ and 1280 cm^−1^ 35 validated qualitatively the high degree of polymerization as calculated from FTIR. However, quantification was not possible with this technique, since the polymers were already too long (for further information see Section 4 in the Supporting Information).^32^

**Figure 1:**
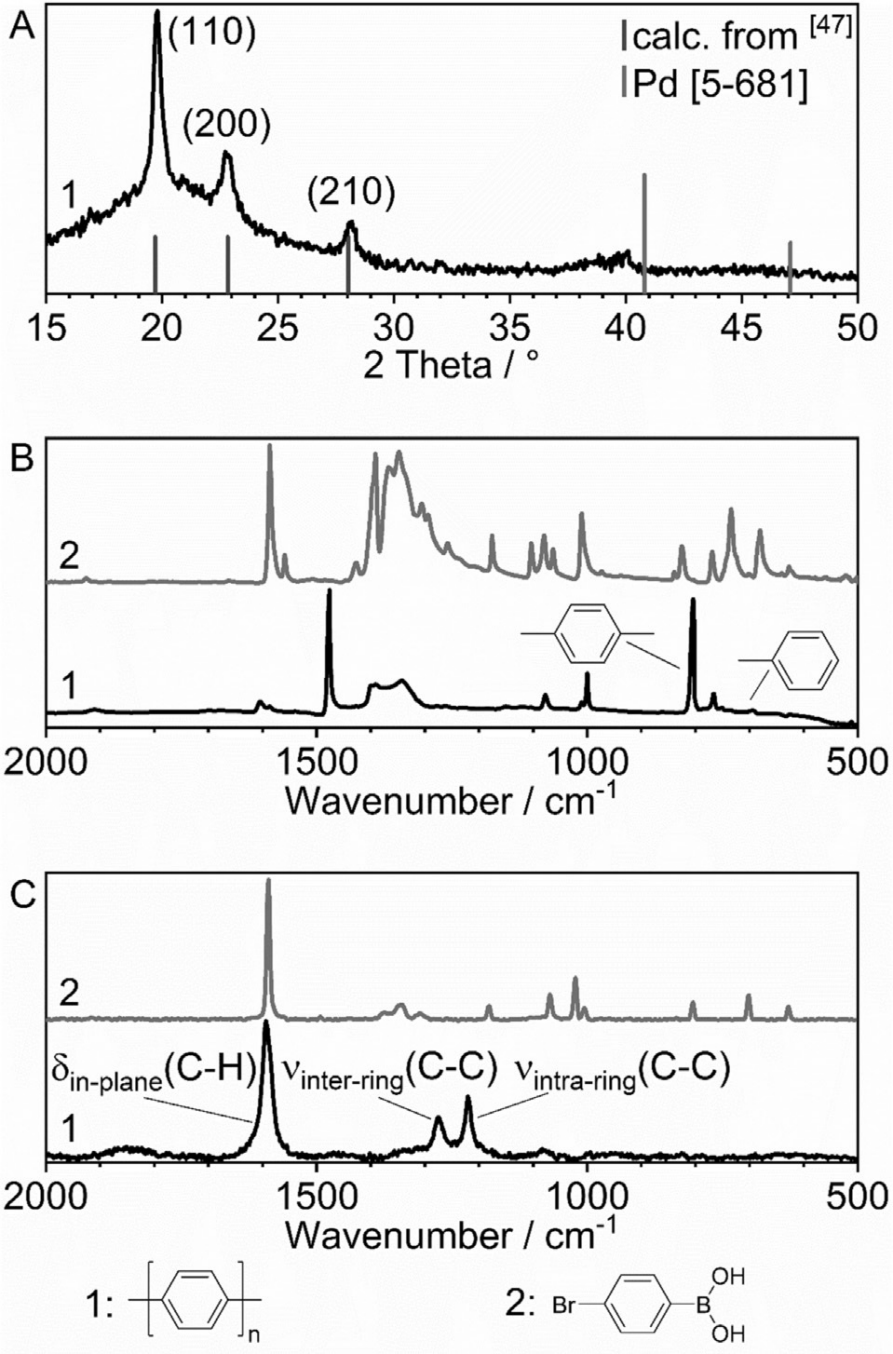
Spectra of sample PPP_PBM_‐6 (1) and the respective monomer (2). A: PXRD pattern of polymer, reference calculated from ref. 47. B: FTIR spectra of monomer and polymer. C: Raman spectra of monomer and polymer.

The samples PPP_PBM_‐1, ‐2, ‐3, ‐6 were milled with ZrO_2_, steel, tungsten carbide (WC), and Si_3_N_4_ milling balls/vessels, respectively. The higher density of the milling equipment resulted in higher yield but lower DP after 4 h milling (Table 1). The higher density and thus higher mass of the milling balls correspond to an increased kinetic energy during ball impacts (*E*_kin_=0.5 mv^2^), which increased the conversion efficiency of the polymerization. In turn, a higher ball impact led to a fracturing of growing polymer chains, resulting in a lower DP (see fracturing tests, Section 1.2 in the Supporting Information).^36^, ^37^

The highest DP was achieved using milling equipment made of Si_3_N_4_, so we continued to use it and investigated the influence of the milling time (PPP_PBM_‐4 to ‐8 in Table 1) on yield and DP. Going from 2 to 5 h of milling did not change the yield drastically, while after 6 h the yield increased to 34 %. On the other hand, the DP reached a maximum of 56 after 4 h (PPP_PBM_‐6, characterization shown in Figure 1), with further milling leading to fracturing and lower DP.

Subsequently, we reduced the amount of Pd black catalyst. With 2.3 mol % (PPP_PBM_‐9) the yield was nearly the same, but with 0.5 mol %, it was drastically reduced to 5 % (PPP_PBM_‐10). The amount of catalyst did not affect the DP, meaning that the equilibrium of polymer chain growth and fracture was mainly dependent on the density of the milling balls as mentioned above (Section 2 in the Supporting Information; considerations about mechanism in Section 7). Thereafter, we examine the halide function of the monomer due to its known influence in Suzuki couplings. PPP was not obtained with 4‐chlorophenylboronic acid (PPP_PBM_‐11), whereas 4‐iodophenylboronic acid gave the highest yield (66 %) and DP (79) (Table 1). The literature supports the observed trend of increasing reactivity on going from chloride to bromide and iodide.^22^, ^38^ For further experiments, we thus continued with 4‐iodophenylboronic acid as the monomer.

To verify the catalytic nature of the reaction, we also performed the reaction without Pd catalyst or with the base replaced by NaCl as an inert bulking material. In both cases, no product was formed, confirming the need for a catalyst and a base in this reaction.

In the third step, we tested Pd milling balls as the catalyst. We had to change the milling setup since using 22 Pd milling balls, as in all other PBM experiments, was too expensive. As an alternative approach, we used a mixer ball mill (MM). Recently, several publications have shown how a Suzuki coupling reaction can be transferred from PBM to MM without affecting the reaction outcome.^39^, ^40^, ^41^ As additional evidence, we first showed that yield and DP of two reference materials synthesized by MM with Pd(OAc)_2_ (PPP_MM_‐Ref‐1) and Pd black (PPP_MM_‐Ref‐2) as catalysts were comparable to the results obtained in a PBM with 4‐bromophenylboronic acid (PPP_PBM_‐Ref and PPP_PBM_‐1) as well as with Pd black catalyst and 4‐iodophenylboronic acid (PPP_PBM_‐12) (Table 2). However, in the case of MM longer milling time was necessary due to the lower energy input and a different mixing regime compared to PBM.

**Table 2:** Poly(*para*‐phenylene) obtained by mechanochemical Suzuki polymerization of 4‐iodophenylboronic acid in a mixer ball mill using Pd(OAc)_2_, Pd black, and Pd milling balls as catalysts.

	Sample code	Vessel/ ball material	Reaction time [h]	Amount of catalyst	DP	Yield of insoluble polymer [%]^[a]^
Reference MM	PPP_MM_‐Ref‐1	ZrO_2_	8	4.7 mol % Pd(OAc)_2_	50	50
	PPP_MM_‐Ref‐2	ZrO_2_	8	4.7 mol % Pd black	52	6
Pd balls/MM	PPP_MM_‐1	ZrO_2_/Pd	8	1 Pd ball	115	6
	PPP_MM_‐2	ZrO_2_/Pd	8	2 Pd balls	99	31
Leaching	PPP_MM_‐3	ZrO_2_/Pd	8	2 Pd balls	199^[b]^	18^[b]^
In situ Raman	PPP_MM_‐4	PMMA/ZrO_2_	8	4.7 mol % Pd black	123	100
	PPP_MM_‐5	PMMA/Pd	14	2 Pd balls	118	50

DP and yield measured five times with a standard deviation of ±5 % [a] Yield calculated from the mass of insoluble polymer after washing with water, 10 wt % HCl, ethanol, and acetone. In the organic washing solutions, smaller oligomers could be detected (see Section 1.2 in the Supporting Information). [b] DP and yield measured three times, with a standard deviation of ±15 %. Densities of the milling materials and weight of the milling balls: ZrO_2_ 5.7 g cm^−3^, 3.19±0.05 g, Pd 12.0 g cm^−3^, 3.6 g.

For direct mechanocatalysis, the standard synthesis in the MM involved 496 mg (2.00 mmol) of 4‐iodophenylboronic acid and 2.504 g (18.12 mmol) of K_2_CO_3_ with one or two palladium milling balls (10 mm diameter) in a 25 mL ZrO_2_ milling vessel. The reaction mixture was milled at 30 Hz (for characterization see Section 3 in the Supporting Information).

The reaction with one Pd milling ball (3.6 g) resulted in a low yield (6 %) but a high DP (115) (PPP_MM_‐1, see Table 2). The high energy impact with one milling ball had a high conversion efficiency but only a few possibly reactive collisions occurred. Also, inefficient mixing of the reaction mixture with one ball may have caused the poor yield. Adding a second milling ball (PPP_MM_‐2) improved the mixing and increased the number of possible reactive collisions but decreased the average velocity and therefore the impact energy of the milling balls because of the reduced free path. As a result, the yield increased (31 %) to a level closer to that of PPP_PBM_‐1, while the DP of 99 was comparable to that obtained with one Pd milling ball. As this setup gave the best results so far, we repeated approach PPP_MM_‐2 another two times, finding a deviation in DP of ±5 % and in yield of ±2 %. This also addresses a good reproducibility of the direct mechanocatalytic Suzuki polymerization.

In order to establish the heterogeneous nature of the reaction protocol, we performed the reaction and removed a 100 mg sample of the reaction mixture every two hours. After 4 h the reaction was briefly stopped and the Pd milling balls were replaced with ZrO_2_ balls of the same size. We then continued the reaction and sampling. While monitoring the reaction, we found that it had significantly slowed down (4 % additional yield over the next 4 hours of milling without Pd balls compared to additional 18 % yield when milled with Pd balls). The continued reaction in the absence of Pd milling balls can be explained by the minor abrasion of Pd from the balls in the first phase of the reaction (for characterization see Section 5 in the Supporting Information). Interestingly, since the ZrO_2_ milling balls are less dense than the Pd milling balls, the polymer fracturing was reduced, resulting in a higher DP (199 compared to 99 for PPP_MM_‐2; for detailed results see Section 5 in the Supporting Information). Moreover, we could not identify any soluble Pd species, supporting a heterogeneous reaction pathway.

In additional experiments, we added common complexation ligands such as triphenylphosphine and 1,5‐cyclooctadiene to the reaction mixture with the aim of Pd stabilization (see Section 5 in the Supporting Information).^42^ Unexpectedly, this did not lead to PPP material. We assume that the ligands were coordinated to the Pd surface of the milling balls and therefore blocked the catalyst sites making them inaccessible for the monomer. This again indicates a heterogeneous reaction at the surface of the Pd.

Finally, we monitored the reaction course by in situ Raman spectroscopy^43^, ^44^ and by in situ synchrotron PXRD^44^, ^45^, ^46^ at the DESY/PETRA III beamline P02.1 (see Section 6 in the Supporting Information). We conducted the reaction according to PPP_MM_‐Ref‐2 and PPP_MM_‐2 in a transparent poly(methyl methacrylate) (PMMA) milling vessel and compared Pd black to Pd milling balls as catalysts.

As seen in Figure 2 B the conversion of the monomer could be followed by the decreasing Raman band of the monomer at 1580 cm^−1^ (Figure 2 B right). The intensity of the main PPP band at 1600 cm^−1^ was too low for following reaction kinetics. Potassium carbonate showed a band at 1060 cm^−1^ that decreased with ongoing reaction. The intensity of the main band from the PMMA milling vessel at 810 cm^−1^ was nearly constant during the whole milling time and therefore suitable as an internal reference.

**Figure 2:**
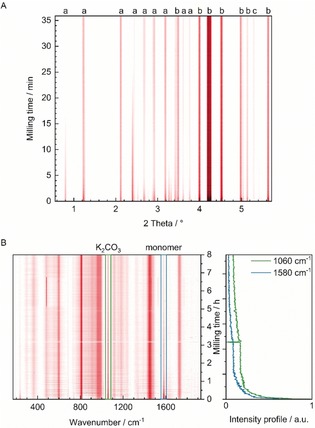
A: In situ PXRD pattern of reaction PPP_MM_‐4. a: commercial monomer containing 4‐iodophenylboronic acid and anhydride. b: K_2_CO_3_ (PDF‐2 [16‐820]). c: Pd (PDF‐2 [5‐681]). B: In situ Raman spectroscopic monitoring of reaction PPP_MM_‐5. At 3 h 10 min the laser had to be switched off briefly such that one acquisition is missing. For experimental details see Section 1.2 in the Supporting Information.

The in situ Raman data for PPP_MM_‐4 (Section 6.2 in the Supporting Information) showed a fast reaction, in which the monomer was completely depleted after a few minutes. The in situ PXRD pattern of PPP_MM_‐4 (Figure 2 A) was consistent with the Raman monitoring and therefore provided qualitative proof of the monomer conversion. the resulting polymers from PPP_MM_‐4 were much longer (DP=123) than those obtained in the ZrO_2_ milling vessel (PPP_MM_‐Ref‐2, 6 %, DP=52) with significantly higher yield (100 % compared to 6 %).

According to the in situ Raman data, the reaction proceeded slower with Pd milling balls (PPP_MM_‐5, Figure 2 B). After 2 h of milling, the monomer was almost fully converted, whereas the yield of insoluble polymer was much lower, indicating the formation of smaller oligomers that were soluble in ethanol or acetone. This was supported by GCMS analysis of organic washing solutions in which traces of iodobenzene, iodobiphenyl, and iodoterphenyl were identified (see Section 1.2 for detailed information). Comparing PPP_MM_‐5 with PPP_MM_‐2, the yield and DP of insoluble polymer increased from 31 % to 50 % and from 99 to 118, respectively (Table 2).

In the end, the softer PMMA milling vessel seemed more suitable for the reaction, since the respective samples gave the highest yield, whereas even the light but hard Si_3_N_4_ milling material with the higher energy input in PBM fractured the PPP significantly. Also, the total abrasion of Pd milling balls in the PMMA vessel was nearly a third (120 mg) of that of PPP_MM_‐2 (ZrO_2_ vessel, 290 mg), yet gave a higher yield. Again, this indicated a heterogeneous reaction at the surface of the milling ball without the need of a certain amount of abraded Pd.^49^ Lastly, the overall results showed a decreased reaction velocity on going from Pd(OAc)_2_ to Pd black and the Pd milling ball catalyst. Longer reaction times led to the fracturing of the formed polymer chains when hard milling equipment, such as ZrO_2_ and Pd, was used, whereas in soft PMMA milling vessel high yields and DPs could be achieved.^50^

In summary, the solvent‐free environment of a ball mill makes it possible to directly use palladium milling equipment or Pd black catalyst, instead of conventional Pd^II^ salts or Pd complexes, as we have shown here for the Suzuki polymerization of *para*‐substituted phenylboronic acids to produce poly(*para*‐phenylene). With 4‐iodophenylboronic acid as the monomer, a good yield and high DP were achieved in PBM using Si_3_N_4_ milling material, while full conversion to long‐chain polymers was obtained in MM using a softer PMMA vessel. In addition, the DPs achievable by this method surpassed those obtained by solution or electrochemical processes, which is beneficial for PPP application in opto‐ and microelectronics. In situ Raman and PXRD investigations were used to monitor the conversion of monomer. Our results indicate a most likely heterogeneous reaction, which was not improved by using established ligands from solution‐based homogeneous procedures.

We also expect the concept of “direct mechanocatalysis” presented here will be used in other palladium‐catalyzed cross‐coupling reactions under solvent‐free conditions beyond the Suzuki cross‐coupling.

## Conflict of interest

The authors declare no conflict of interest.

## Supporting information

As a service to our authors and readers, this journal provides supporting information supplied by the authors. Such materials are peer reviewed and may be re‐organized for online delivery, but are not copy‐edited or typeset. Technical support issues arising from supporting information (other than missing files) should be addressed to the authors.

Supplementary
