# Supplementary material for: Direct Mechanocatalysis: Palladium as Milling Media and Catalyst in the Mechanochemical Suzuki Polymerization
Source: Angew Chem Int Ed Engl. 2019 Nov 7;58(52):18942–7. doi: 10.1002/anie.201911356 (PMC6972522; doi:10.1002/anie.201911356)
Supplement: Supplementary file 1 — Supplementary [file ANIE-58-18942-s001.pdf]

Angewandte Chemie
Eine Zeitschrift der Gesellschaft Deutscher Chemiker

Supporting Information

Direct Mechanocatalysis: Palladium as Milling Media and Catalyst in the Mechanochemical Suzuki Polymerization

*Christian G. Vogt, Sven Grätz, Stipe Lukin, Ivan Halasz, Martin Etter, Jack D. Evans, and Lars Borchardt**

anie_201911356_sm_miscellaneous_information.pdf

Author Contributions

C.V. Investigation: Lead; Methodology: Lead; Visualization: Lead; Writing - Original Draft: Equal; Writing - Review & Editing: Equal
S.G. Conceptualization: Equal; Data curation: Supporting; Investigation: Supporting; Methodology: Equal; Supervision: Supporting; Visualization: Supporting; Writing - Original Draft: Equal; Writing - Review & Editing: Equal
S.L. Data curation: Supporting; Investigation: Supporting; Resources: Supporting; Software: Supporting; Visualization: Supporting; Writing - Original Draft: Supporting; Writing - Review & Editing: Equal
I.H. Supervision: Supporting; Writing - Original Draft: Supporting; Writing - Review & Editing: Supporting
M.E. Resources: Equal; Software: Equal; Writing - Review & Editing: Supporting
J.E. Data curation: Supporting; Methodology: Equal; Software: Supporting; Writing - Review & Editing: Supporting
L.B. Conceptualization: Lead; Data curation: Equal; Methodology: Equal; Supervision: Lead; Writing - Original Draft: Equal; Writing - Review & Editing: Lead.

Table of Contents

1. Experimental Section 2
1.1. Used Equipment and Characterization Techniques 2
1.2. Synthetic Procedures 3
2. Characterization of Samples from Planetary Ball Mill 4
3. Characterization of Samples from Mixer Mill 6
4. Estimation of Polymerization Length from Raman Spectra 8
5. Leaching Test 8
6. In situ Experiments 10
6.1. Reference Raman Spectra 10
6.2. PPP_{MM}-4 with Pd black Catalyst 11
6.3. PPP_{MM}-5 with Pd Milling Balls 12
7. Considerations about Mechanism 14
8. Green Metrics and Sustainability 14
9. Mechanosynthesis of 1,3,5-Triphenylbenzene 17
References 18

SUPPORTING INFORMATION

1. Experimental Section

1.1. Used Equipment and Characterization Techniques

General. Palladium(II) acetate, 4-iodophenylboronic acid and 4-chlorophenylboronic acid were purchased from TCI-Chemicals. 4-Bromophenylboronic acid and palladium black were purchased from abcr GmbH. Potassium carbonate was purchased from Grüssing. Phenylboronic acid was purchased from Frontier Scientific. 1,3,5-Tribromobenzene was purchased from Alfa Aesar. All chemicals were used without further purification.

Powder X-ray diffraction (PXRD) patterns were collected in transmission geometry (DECTRIS MYTHEN 1K detector) with a STOE STADI P diffractometer operated at 40 kV and 40 mA with a Ge Monochromator using Cu-Kα1 radiation. For estimating particle sizes of Pd the Scherrer equation (1) was used with Scherrer constant K = 0.89 and λ = 0.15406 nm. FWHM was determined by peak integration of the (111) reflex with highest intensity (40° 2θ) using Origin 2018G software.

$$D = \frac{K\lambda}{FWHM \cdot cos\theta} \qquad \text{Equation (1)}$$

Fourier-transform infrared spectroscopy (FT-IR) was carried out on a BRUKER Vertex 70 with a Specac Golden Gate ATR unit. A resolution of 2 cm^{-1} was utilized and the resulting spectra were treated with ATR-correction by the OPUS 6.5 software. Each sample was measured five times and the average of the integrals was taken in order to reduce effects caused by inhomogeneities. **Degree of Polymerisation** (DP) was calculated from FT-IR spectra with the principal absorption band at 805 cm^{-1} from *p*-substituted phenyl rings and the smaller band at 690 cm^{-1} attributed to the vibration of terminal phenyl rings. Using the intensities of these bands in equation (2) gives the DP.

$$DP = 2 \cdot \frac{I_{805}}{I_{690}} + 2 \qquad \text{Equation (2)}$$

Raman spectra were obtained using a RENISHAW inVia Qontor Raman microscope with 50x object (NA = 0.50, 8.2 mm free working distance). The wavelength for the measurement was 532 nm with 1 to 5% laser power dependent on the sample. The exposure time was 10 s, 3 accumulations were collected. For in situ monitoring a fiber optic was used with 5x object (NA = 0.12, 14 mm free working distance). The object was adjusted to the milling vessel to ensure measurement of the reaction mixture. The spectra were collected from 6 accumulations with 10 s exposure time (100% laser power) each without an interval between the measurements. All obtained spectra were treated with baseline correction and cosmic ray removal using WiRe 5.1 software.

Elemental analyses were carried out on a vario MICRO cube Elemental Analyzer by Elementar Analysatorsysteme GmbH in CHNS modus.

Gas chromatography-mass spectrometry (GC-MS) analyses were carried out with a Shimadzu GCMS-QP5000 using electron ionization at 200 °C injection temperature. The used column was a Trajan BPX5 (length 30 m, inner diameter 0.25 mm and film thickness 0.25 µm). The interface temperature was 300 °C. A temperature program for the column was utilized from 50 °C (1 min hold) to 200 °C (30 K min^{-1}) and further on to 360 °C (15 K min^{-1}, held for 10 min).

Scanning electron microscopy (SEM) images were obtained using a Hitachi SU8020 SEM equipped with a secondary electron (SE) detector. Prior to the measurement the samples were prepared on an adhesive carbon pad and sputtered with gold to obtain the necessary electron conductivity.

Milling balls. Zirconium dioxide (Type ZY-S) milling balls with a diameter of 10 mm were purchased from Sigmund Lindner GmbH. The average weight of one milling ball is 3.19 ± 0.05 g. Tempered steel (1.4125, AISI 440C) milling balls with a diameter of 10 mm were purchased from TIS Wälzkörpertechnologie GmbH. The average weight of one milling ball is 4.02 ± 0.02 g. Tungsten carbide (YG6X, G10 surface) milling balls with a diameter of 10 mm were purchased from Zhuzhou Good Cemented Carbide Co., Ltd. The average weight of one milling ball is 7.20 ± 0.26 g. The WC balls measured had already been used to a different degree, this explains the big variations (standard deviation) in the mass per ball. Silicon nitride (Type SNCB5) milling balls with a diameter of 10 mm were purchased from Fritsch GmbH. The average weight of one milling ball is 1.94 ± 0.03 g. Palladium milling balls with a diameter of about 9 mm were purchased from SMT Metalle Wimmer. They were prepared by arc melting and therefore not of perfect spherical shape. The weight of the milling balls was about 3.6 g and reduced over the experiments due to abrasion.

1.2. Synthetic Procedures

Mechanosynthesis of PPP in planetary ball mill

For the experiments in planetary ball mill (PBM) 4-bromophenylboronic acid (1.454 g, 7.23 mmol), palladium black (36 mg, 4.7 mol%) and potassium carbonate (8.510 g, 61.58 mmol) were placed together with twenty-two 10 mm-diameter grinding balls in a 45 mL grinding jar. The mixture was milled for 2 to 6 h at 800 rpm in a Fritsch Pulverisette 7 premium line planetary ball mill. The samples were washed with water, 10 wt% HCl, ethanol and acetone and afterwards dried over night at 80 °C.

Mechanosynthesis of PPP in mixer mill

The experiments in mixer mill (MM) were conducted with 4-iodophenylboronic acid (496 mg, 2.00 mmol) and potassium carbonate (2.504 g, 18.12 mmol) were placed in a 25 mL zirconium oxide grinding jar together with two palladium balls of about 10 mm diameter. The mixture was milled for 8 h in a Retsch MM400 mixer mill at 30 Hz. For reference experiments 4.7 mol% catalyst (palladium(II) acetate or palladium black) were added and the palladium balls were replaced by zirconium oxide balls. The samples were washed with water, 10 wt% HCl, ethanol and acetone and afterwards dried over night at 80 °C.

Yield calculation and conversion

The yield of insoluble polymer was calculated from the weight of the sample after washing and drying. The mass of Pd in the sample (Pd black deployed or Pd ball abrasion) was subtracted from the sample weight. End groups were not considered.
We examined the organic washing solution by drying under vacuum and weighing this fraction. The total mass of substance soluble in ethanol and acetone approximately corresponds to the amount of non-reacted monomer calculated from the yield of long chain polymer. Besides remaining monomer, we validated traces of small oligomers (iodobiphenyl, iodoterphenyl) and even deboronated iodobenzene via GC-MS. That shows a higher monomer conversion than the yield denoted. We could not fully determine structure and quantity of the oligomers as well as oligomers with boronic acid functional groups via HPLC-MS. In addition, the widely varying solubility impedes the analysis by this technique.

Fracturing tests

To prove the fracturing of the polymer during the milling we performed experiments milling the synthesized polymers after purification. The first experiment was conducted corresponding to PPP_{PBM}-8, using the standard approach with Si_3N_4 milling material but replacing the monomer 4-bromophenylboronic acid by the insoluble PPP and milling for 6 h in the P7. The second experiment was conducted corresponding to PPP_{MM}-Ref-2 and thus was milled for 8 h with ZrO_2 milling jar and two 10 mm ZrO_2 milling balls in the MM400.The samples were afterwards washed with water, 10 wt% HCl, ethanol and acetone and dried over night at 80 °C. For both fracturing tests a drastically degradation of polymer was observed. The initial of the milled polymer of 120 was reduced to 39 after 6 h milling in PBM and similarly to 36 after 8 h milling in MM. This clearly proves the mentioned degradation effects.

Leaching test

To study the heterogeneity of the reaction in sample PPP_{MM}-3 a standard reaction protocol was conducted in which the Pd balls were replaced by two ZrO_2 balls after half the reaction time. Every two hours 100 mg reaction mixture were taken from the grinding jar to determine yield and DP.

In situ Raman spectroscopic and in situ PXRD investigation

In literature is shown that mechanochemical reactions can by monitored in situ by Raman spectroscopy[1,2] and synchrotron PXRD.[2–4] We realized the same approach by milling 4-iodophenylboronic (496 mg, 2.00 mmol) acid with potassium carbonate (2.504 g, 18.12 mmol) and two Pd balls (10 mm, approx. 3.6 g each) in a PMMA ThermoJar milling vessel with temperature tracking from InSolido Technologies (PPP_{MM}-4 and -5).
Sample PPP_{MM}-4 was repeated with in situ synchrotron PXRD. Two-dimensional PXRD images were collected at the DESY/PETRA III beamline P02.1 using a Perkin Elmer XRD 1621 flat panel detector consisting of an amorphous Si sensor equipped with a CsI scintillator (pixel number 2045 x 2048, pixel size 200 x 200 µm²). Diffraction images were subsequently integrated in one-dimensional PXRD patterns with the DAWN Science package[5] using a wavelength $\lambda = 0.020718$ nm. For creating a 2D figure, integrated diffraction patterns were baseline corrected with Sonneveld-Visser algorithm.[6]

2. Characterization of Samples from Planetary Ball Mill

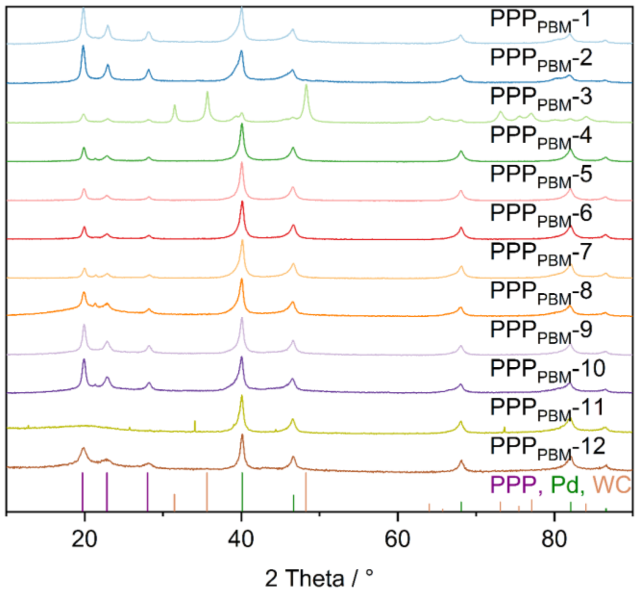

Figure S1. PXRD patterns of the samples synthesized in PBM. Reference for PPP calculated from [7], Pd: PDF-2 [5-681], WC: PDF-2 [25-1047].

Table S1. Peak center θ, FWHM and calculated particle size from PXRD for samples synthesized in PBM.

Sample code	Θ in °	FWHM	D in nm
PPP_{PBM}-1	20.03	0.6079	14
PPP_{PBM}-2	20.00	0.8006	10
PPP_{PBM}-3	20.00	0.6164	14
PPP_{PBM}-4	20.03	0.5761	15
PPP_{PBM}-5	20.03	0.5382	16
PPP_{PBM}-6	20.06	0.5891	14
PPP_{PBM}-7	20.06	0.6134	14
PPP_{PBM}-8	20.03	0.6557	13
PPP_{PBM}-9	20.03	0.5696	15
PPP_{PBM}-10	20.03	0.6252	13
PPP_{PBM}-11	20.06	0.6901	12
PPP_{PBM}-12	20.06	0.5240	16
Pd black	20.01	0.3831	22

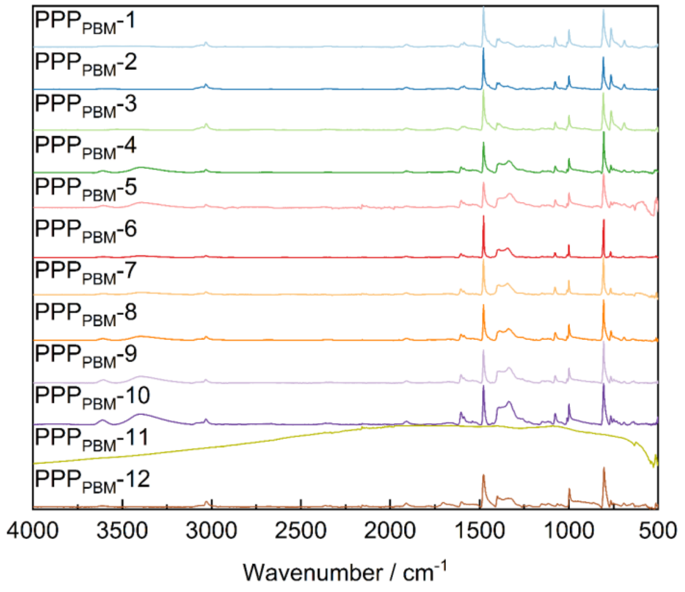

Figure S2. FT-IR spectra of the samples synthesized in PBM.

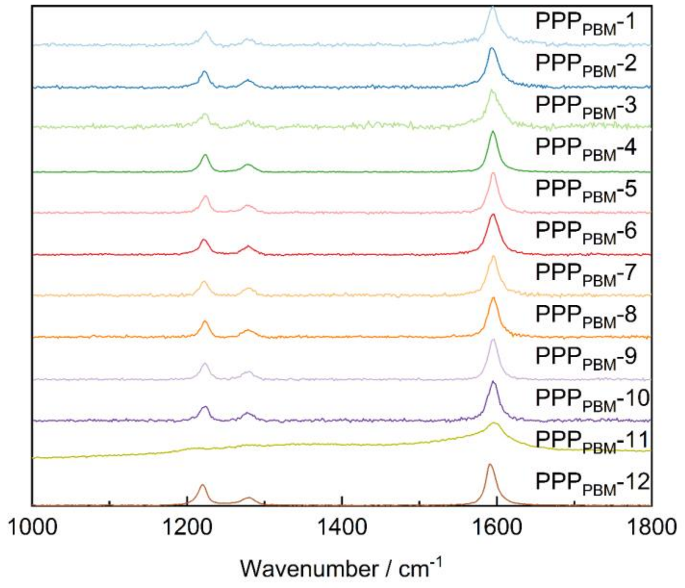

Figure S3. Raman spectra of the samples synthesized in PBM.

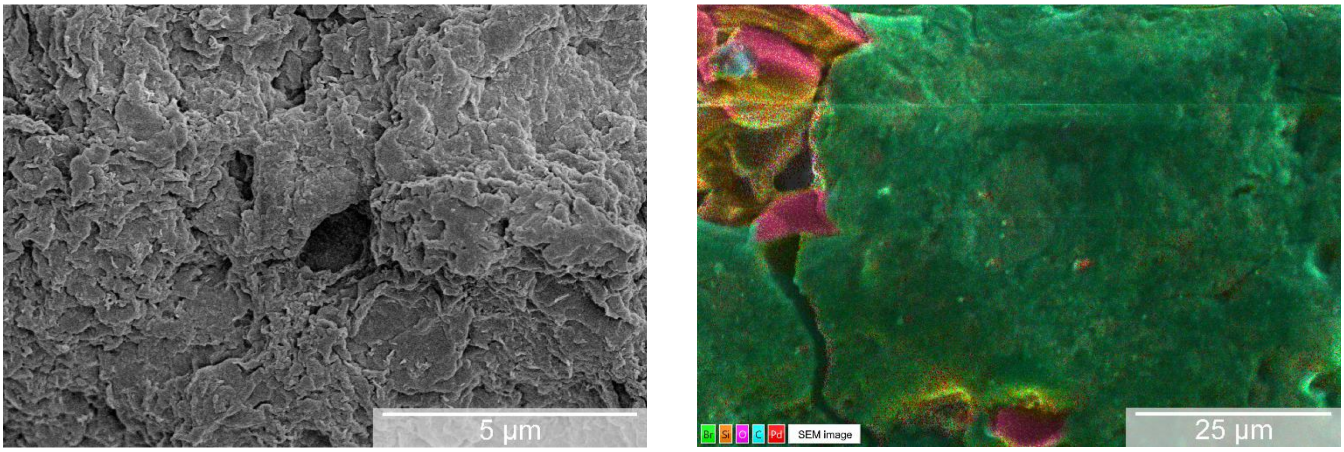

Figure S4. SEM image (left) and EDX mapping (right) exemplarily of sample PPP_{PBM}-6. Pd is homogeneously distributed in the polymer of which the bromide end groups can be seen. Si is abrasion from the frit used for filtration.

Table S2. Elemental analyses results of selected samples synthesized in PBM.

Sample code	C wt%	H wt%	C/H measured	C/H calc.
PPP_{PBM}-1	61.90	3.435	1.512	1.5
PPP_{PBM}-2	70.97	4.030	1.477	1.5
PPP_{PBM}-3	70.02	3.944	1.490	1.5
PPP_{PBM}-6	57.81	3.224	1.505	1.5

The results from elemental analysis (Table S2) are in good accordance with the theoretical calculated values for poly(*para*-phenylene) taking end groups not into account.

In samples PPP_{PBM}-9 and PPP_{PBM}-10 the amount of Pd black catalyst was reduced to 2.3 and 0.5 mol% respectively. As a result, the DP (PPP_{PBM}-9: 55, PPP_{PBM}-10: 48) was not affected significantly compared to PPP_{PBM}-6 (56). This could give a hint on the polymerization mechanism which is expected to be a polycondensation as type of a step-growth polymerization. In the case of a chain-growth polymerization the DP should have increased with reduced catalyst amount. However, the chain fracturing as mentioned in the main text plays a role. This indicates the often observed phenomenon of changing reaction mechanisms in mechanochemistry compared to classical synthesis.

3. Characterization of Samples from Mixer Mill

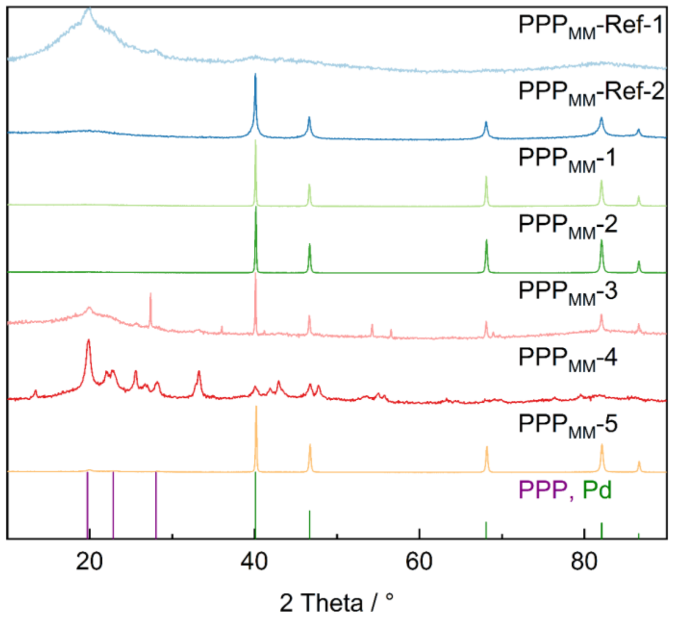

Table S3. Peak center θ, FWHM and calculated particle size from PXRD for samples synthesized in MM.

Sample code	Θ in °	FWHM	D in nm
PPP_{MM}-Ref-2	20.03	0.2014	42
PPP_{MM}-1	20.05	0.1234	68
PPP_{MM}-2	20.07	0.1458	57
PPP_{MM}-3	20.05	0.1536	54
PPP_{MM}-4	20.05	0.1441	58
PPP_{MM}-5	20.09	0.1525	55

Figure S5. PXRD patterns of samples synthesized in MM. Reference PPP calculated from [7], Pd: PDF-2 [5-681].

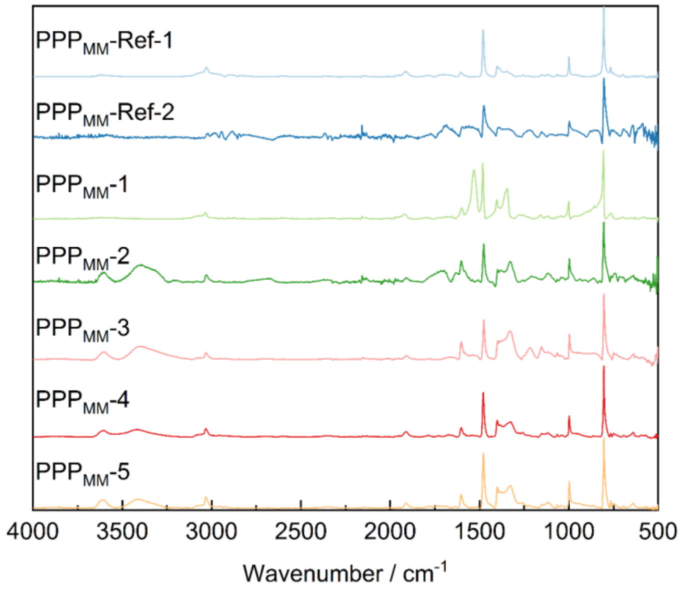

Figure S6. FT-IR spectra of samples synthesized in MM.

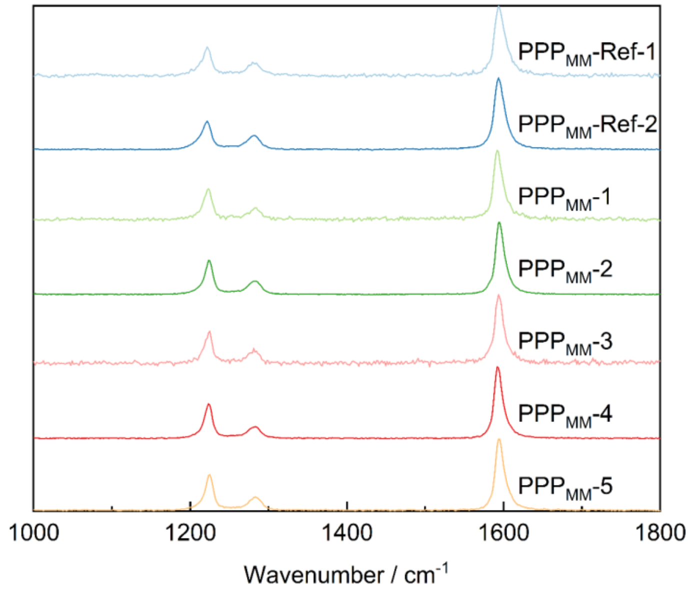

Figure S7. Raman spectra of samples synthesized in MM.

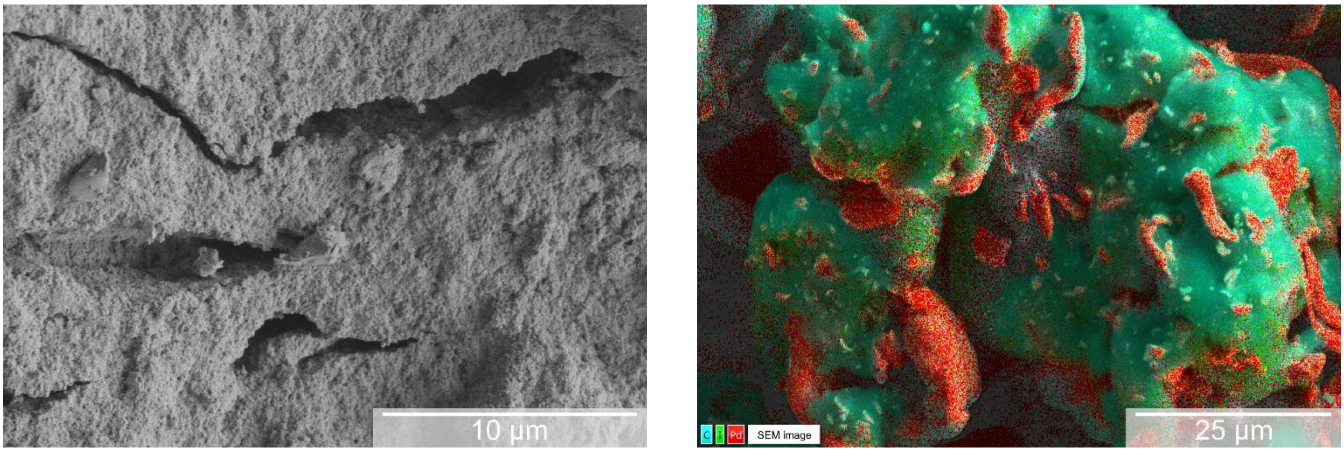

Figure S8. SEM image (left) and EDX mapping (right) exemplarily of sample PPP$_{MM}$-2. Pd is incorporated in bigger particles compared to PPP$_{PBM}$-6 above. End groups (here iodide) are also present.

4. Estimation of Polymerization Length from Raman Spectra

The specific Raman bands of PPP at 1220 cm^{-1} ($\nu_{intra-ring}$(C-C)) and 1280 cm^{-1} ($\nu_{inter-ring}$(C-C)) can be used to determine the chain length of PPP.[8] Actually, the intensity ratio $I_{1280} : I_{1220}$ decreases with increasing number of phenyl rings (Figure S9) from ≈22 (biphenyl) to ≈1 (*p*-sexiphenyl). All our samples show lower values about 0.5, proving the high DP calculated from FT-IR spectra (Figure S9). In contrast, quantification of the polymerization length via Raman is not possible with our data. To obtain the intensity ratio $I_{1280} : I_{1220}$ we measured the intensities of these peaks by fitting a Lorentzian function to the collected Raman spectra.[9]

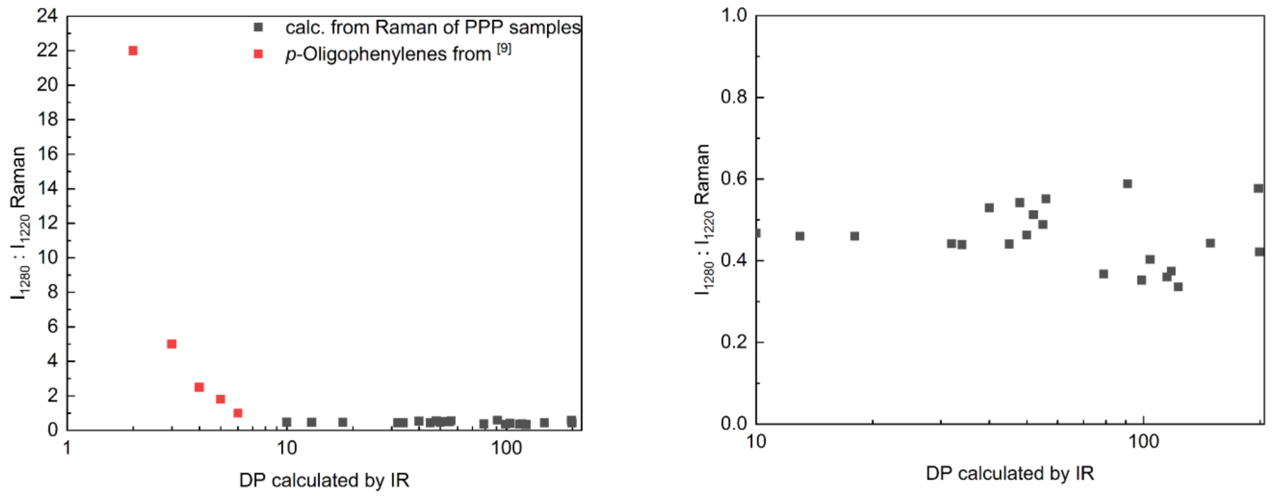

Figure S9. Intensity ratio obtained by Raman spectra correlated to chain length values from literature[9] (left) and DP calculated by FT-IR spectra (left, right extended view). Logarithmic x-scale was chosen just for better visibility.

5. Leaching Test

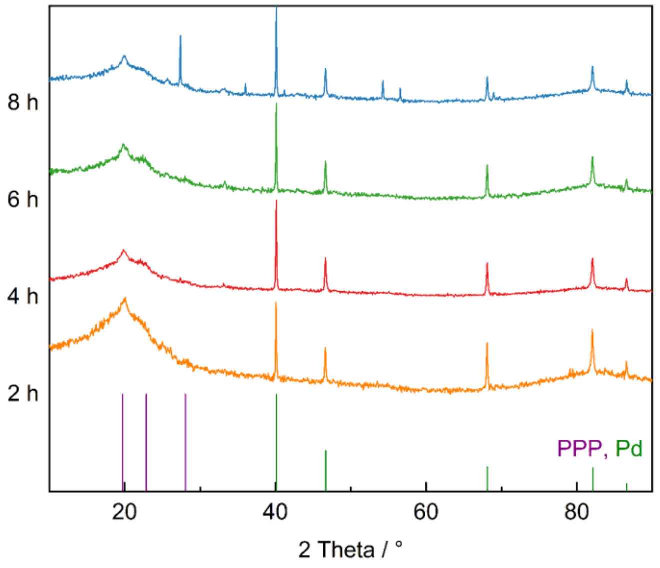

Figure S10. PXRD patterns of sample from PPP_{MM}-3 taken after different milling times. Reference PPP calculated from [7], Pd: PDF-2 [5-681].

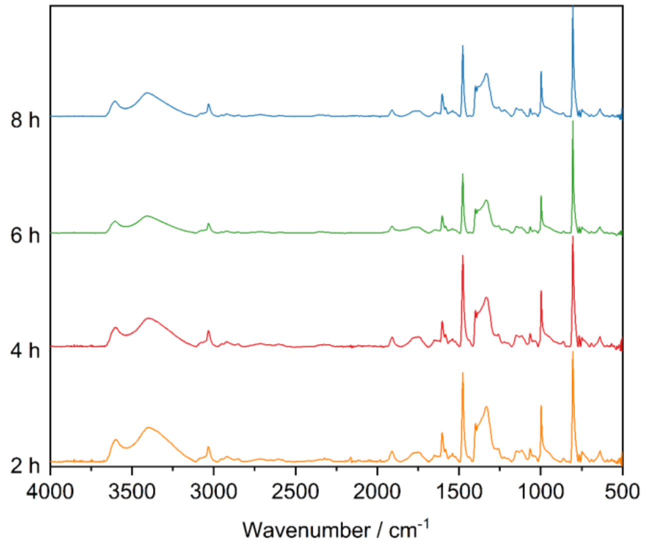

Figure S11. FT-IR spectra of sample from PPP_{MM}-3 taken after different milling times

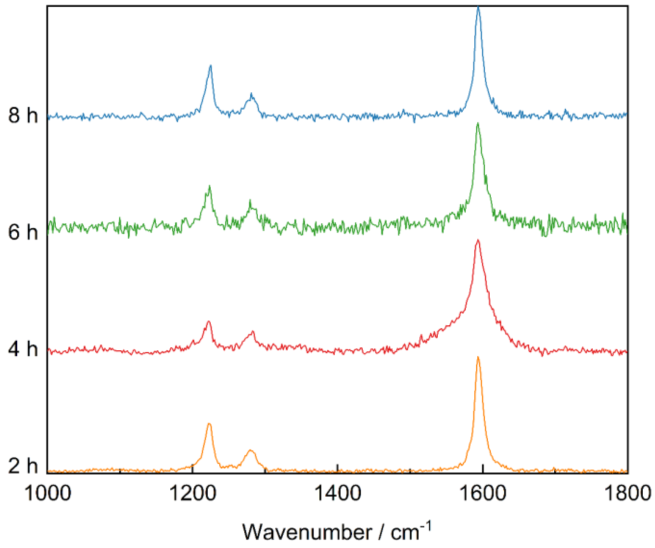

Figure S12. Raman spectra of sample from PPP_{MM}-3 taken after different milling times.

We determined the mass of the Pd milling balls after every reaction to evaluate the amount of abraded Pd. Experiment PPP_{MM}-2 with two Pd balls showed loss of 290 mg in sum for the two Pd balls. Interestingly, the experiment PPP_{MM}-1 with one Pd ball had 590 mg loss in ball mass. This difference could be expected since one ball has higher impact energy and thus the material is probably more prone to abrasion. Average particle sizes were determined from PXRD patterns by Scherrer method (ESI section 1.1). We found an average particle size of 57 nm for PPP_{MM}-2, in contrast to lower values found in PBM experiments with Pd black catalyst (Table S1). These findings are supported by SEM images (see Figures S4 and S8).

Table S4. Poly(*para*-phenylene) samples from PPP_{MM}-3 after different milling times.

Reaction time [h]	Vessel/ball material	Amount of catalyst	DP[b]	Yield insoluble polymer [%][a][b]	Θ in °	FWHM	D in nm
2	ZrO_2/Pd	2 Pd balls	149	6	0.1260	20.03	66
4	ZrO_2/Pd	2 Pd balls	91	14	0.1296	20.05	65
6	ZrO_2	/	198	23	0.1235	20.04	68
8	ZrO_2	/	199	18	0.1184	20.05	71

[a] Yield calculated from the mass of insoluble polymer after washing with water, 10 wt% HCl, ethanol and acetone. In the organic washing solutions smaller oligomers could be validated (see ESI 1.2). [b] Please note that DP and yield could not be determined exactly due to low sample amounts of less than 5 mg.

In two additional experiments we added the common complexation ligands triphenylphosphine and 1,5-cyclooctadiene to the reaction (with the aim of Pd stabilization). This did not lead to PPP material. We assume that the ligands are coordinated to the Pd surface and therefore block the catalyst sites making them inaccessible for the monomer. This is supported by literature, showing for solvent-free mechanochemical Sonogashira reaction that no in situ complex formation occurs when $Pd(OAc)_2$ catalyst is used adding triphenylphosphine.[10]

6. In situ Experiments

6.1. Reference Raman Spectra

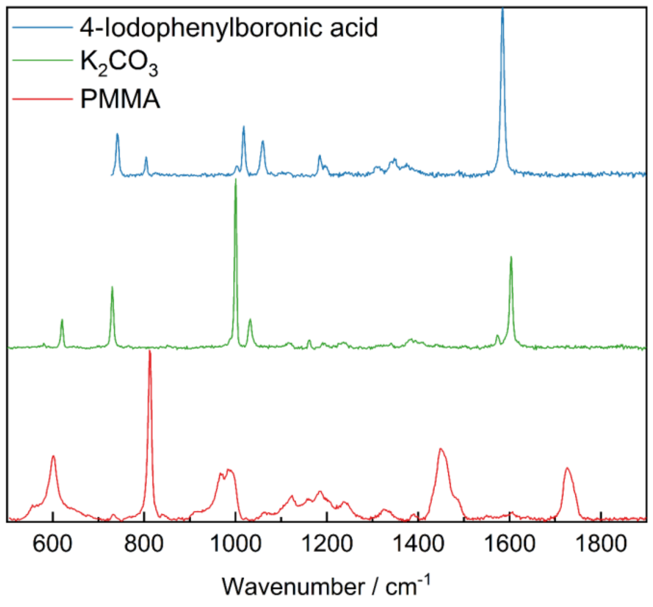

Figure S13. Reference Raman spectra of the compounds from in situ Raman investigations.

6.2. PPP$_{MM}$-4 with Pd black Catalyst

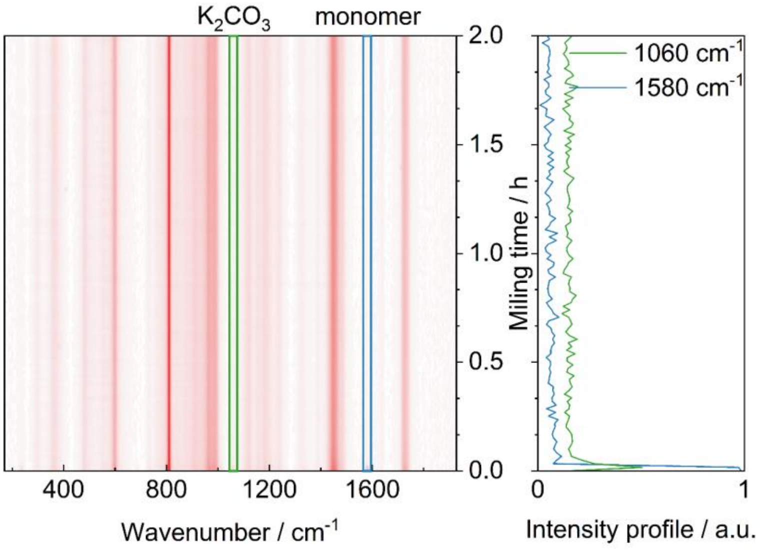

Figure S14. In situ Raman of sample PPP$_{MM}$-4 synthesized with Pd black catalyst (left) with intensity profiles for 4-Iodophenylboronic acid monomer and K_2CO_3 during milling time (right). The main band of PMMA at 810 cm^{-1} does not change significantly.

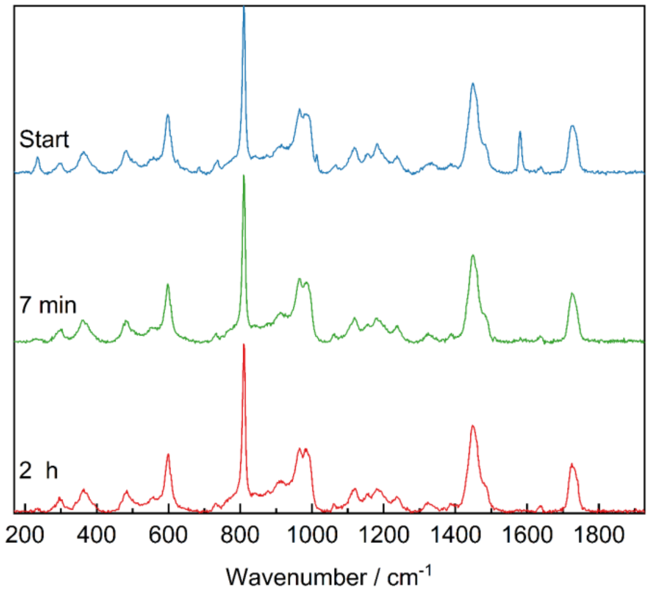

Figure S15. Raman spectra extracted from in situ data at the start, after 7 min and 2 h of milling. The decreasing bands of monomer at 236 cm^{-1} and 1580 cm^{-1}, and K_2CO_3 at 1060 cm^{-1} could be observed.

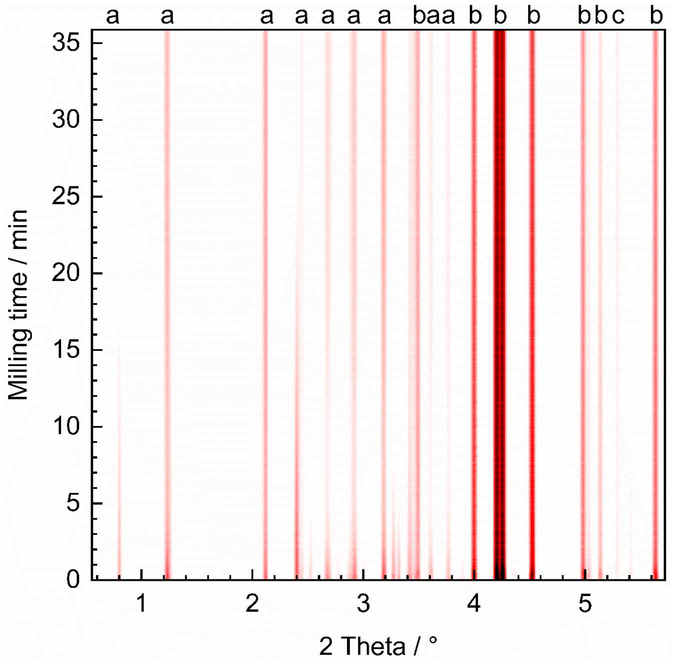

Figure S16. In situ synchrotron PXRD of sample PPP_{MM}-4 synthesized with Pd black catalyst. a: Reflexes of commercial monomer, containing 4-iodophenylboronic acid and anhydride. b: Reflexes of K_2CO_3 (PDF-2 [16-820]). c: Reflex of Pd (PDF-2 [5-681]).

6.3. PPP_{MM}-5 with Pd Milling Balls

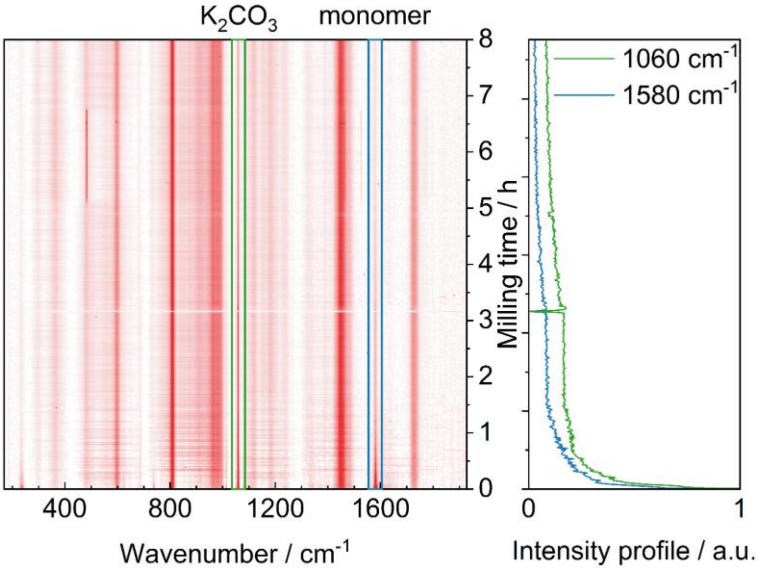

Figure S17. In situ Raman of sample PPP_{MM}-5 synthesized with 2 Pd balls as catalyst.

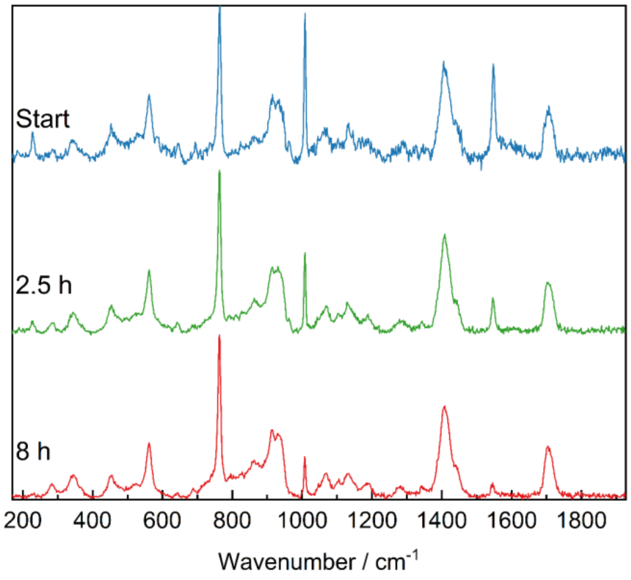

Figure S18. Raman spectra extracted from in situ data at the start, after 2.5 h and 8 h of milling. The decreasing bands of monomer at 236 cm^{-1} and 1580 cm^{-1}, and K_2CO_3 at 1060 cm^{-1} could be observed.

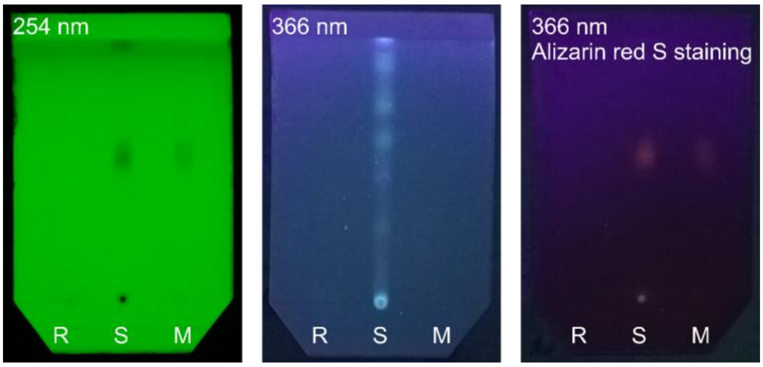

Figure S19. Thin layer chromatography of organic solvent fraction from washing sample PPP_{MM}-5 (S), monomer 4-iodophenylboronic acid (M) and as reference iodobenzene (R). As eluent *n*-pentane:ethyl acetate (60:40) was used. Under 254 nm light three spots are visible, indicating iodobenzene and remaining monomer. 366 nm light shows a broad variety of substances where oligophenyls may cause the fluorescence. Alizarin red S staining shows a slight yellow/orange fluorescence under UV light with 366 nm selectively for boronic acids, identifying remaining monomer as well as some insoluble boronic compound.[11]

7. Considerations about Mechanism

In samples PPP_{PBM}-9 and PPP_{PBM}-10 the amount of Pd black catalyst was reduced to 2.3 and 0.5 mol% respectively. As a result, the DP (PPP_{PBM}-9: 55, PPP_{PBM}-10: 48) was not affected significantly compared to PPP_{PBM}-6 (56). This observation might indicate a step-growth mechanism for the polymerization reaction. In the case of a chain-growth polymerization the DP should have increased with reduced catalyst amount. In contrast, the results from MM contradict the hypotheses of a step-growth mechanism. Therefore, the reduced DP with increased yield is out of trend and with high DP no unreacted monomer should be found. However, considering our observations of the degrading DP by milling of the polymer (ESI section 1.2) a determination of the mechanism by DP alone is prone to error. In general, we did not determine the polymerization mechanism at this point. Mechanochemical reactions are often supposed to follow radical mechanisms, and radical mechanism in polymerization are mostly found in chai-growth reactions. In the case of this mechanocatalytic Suzuki polymerization one could also speculate about a radical mechanism, but we have no proof for this hypothesis and further investigations are necessary.

8. Green Metrics and Sustainability

Several "green metrics" are a useful tool to evaluate different processes and to quantify their ecological footprint.[12,13] These were calculated in Table S5 in comparison to a solvent-based reaction procedure. In order to compare the reaction procedures itself of solvent-based and mechanochemical approaches the washing procedure was supposed to be the same and therefore not considered.

Table S5. Green metrics of the Suzuki polymerization.

Type	Catalyst	Sample code	Halide function of monomer	Atom economy[a]	Mass intensity[b]	Mass productivity[c]	Reaction time
Solvent-based[d]	$Pd(PPh_3)_4$	P1S[14]	Br	38	156	0.64	48 h
Mechanochemical[15]	$Pd(OAc)_2$	PPP_{PBM}-Ref	Br	38	38.7	2.6	30 min
Mechanochemical	Pd black	PPP_{PBM}-6	Br	38	82.5	1.2	4 h
Mechanochemical	Pd black	PPP_{PBM}-12	I	31	27.8	3.6	4 h
Mechanochemical	Pd milling balls	PPP_{MM}-2	I	31	61.6	1.6	8 h

[a] Atom economy $(AE) = \left(\frac{M(Product)}{\Sigma M(Educts)}\right) x\ 100$. [b] Mass intensity $(MI) = \left(\frac{Total\ mass\ used\ in\ the\ process}{Mass\ of\ product}\right)$. [c] Mass productivity $(MP) = \left(\frac{1}{MI}\right) x\ 100$.
[d] Green metrics for the solvent-based reaction were calculated from [14]. This reaction used an A2B2 approach with 1,4-dibromobenzene and 1,4-benzenediboronic acid as monomers instead of bifunctional monomer (AB) in the mechanochemical reaction. The solvent-based approach additionally was performed under inert conditions and led to a DP of about 40.

To proof the sustainability of our approach we conducted the reaction in PBM with powdery Pd waste instead of Pd black. We obtained PPP materials. With bromide monomer (PPP_{PBM}-13) yield (14%) and DP (51) are comparable to Pd black catalyst (PPP_{PBM}-6). In contrast PPP_{PBM}-14 with iodide monomer shows lower yield (11%) than PPP_{PBM}-12. On the other hand, the DP increases up to 99 maybe due to the slower reaction. In general, it is not necessary to use commercial Pd black since this is a possible way to reuse Pd waste.

Table S6. Additional samples synthesised with powdery Pd waste as catalyst.

	Sample code	Beaker/ball material	Reaction time [h]	Amount of catalyst	DP	Yield insoluble polymer [%][a]	Halide function of monomer
Pd black / PBM	PPP_{PBM}-13	Si_3N_4	4	4.7 mol% Pd waste	51	14	Br
	PPP_{PBM}-14	Si_3N_4	4	4.7 mol% Pd waste	99	11	I

[a] yield calculated from mass of insoluble polymer after washing with water, 10 wt% HCl, ethanol and acetone. Densities of the milling material and weight of the milling balls: Si_3N_4 3.25 g cm^{-3} 1.94 ± 0.03 g.

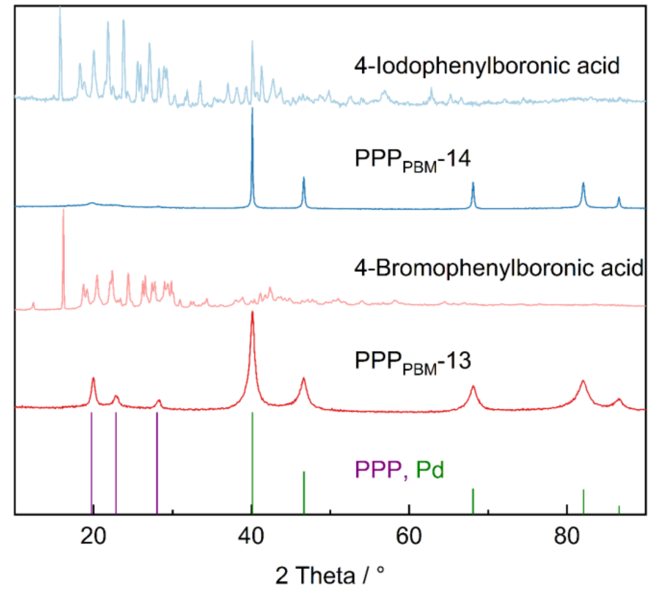

Figure S20. PXRD patterns of samples synthesized with Pd waste in PBM with respective monomer patterns. Reference PPP calculated from [7], Pd: PDF-2 [5-681].

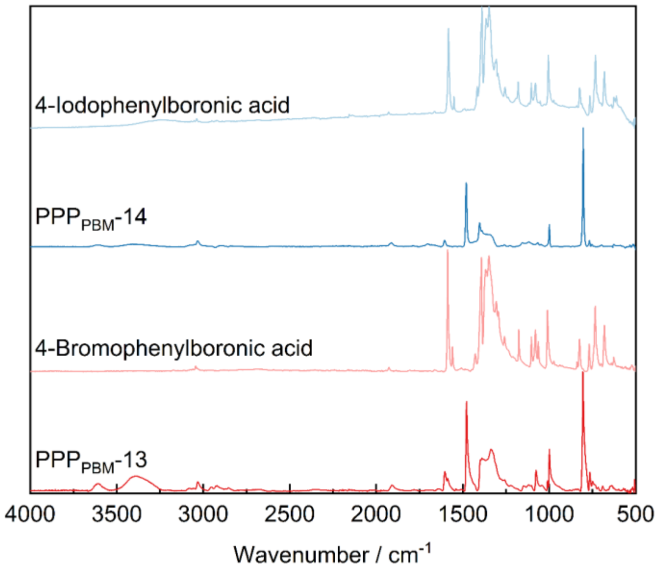

Figure S21. FT-IR-spectra of samples synthesized with Pd waste in PBM with respective monomer spectra.

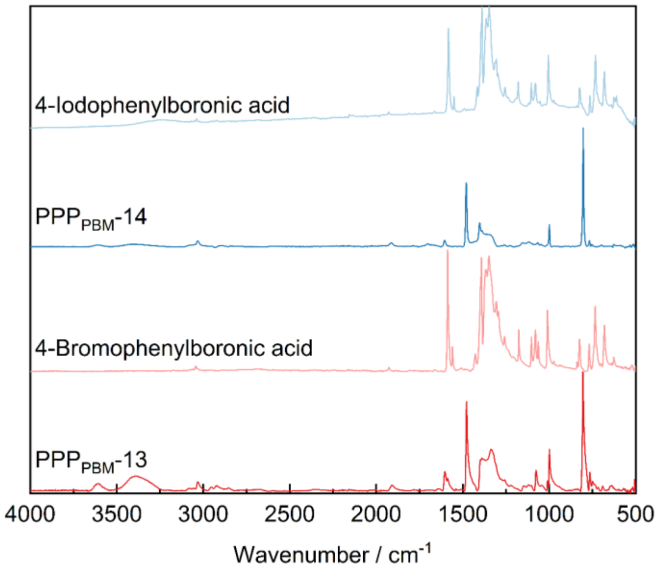

Figure S22. Raman-spectra of samples synthesized with Pd waste in PBM with respective monomer spectra.

Another important factor for greener and sustainable synthesis is the reusability of a catalyst. The reusability of the Pd milling balls by far exceeds that of Pd catalysts in homogeneous catalysis. By now the Pd balls were used for more than 140 h of milling, respectively 20 experiments. This includes pre-tests and unsuccessful approaches that led to high abrasion. Using only optimized conditions will increase the reusability significantly. In Figure S23 the optical change of the Pd balls is displayed on a macroscopic and microscopic scale.

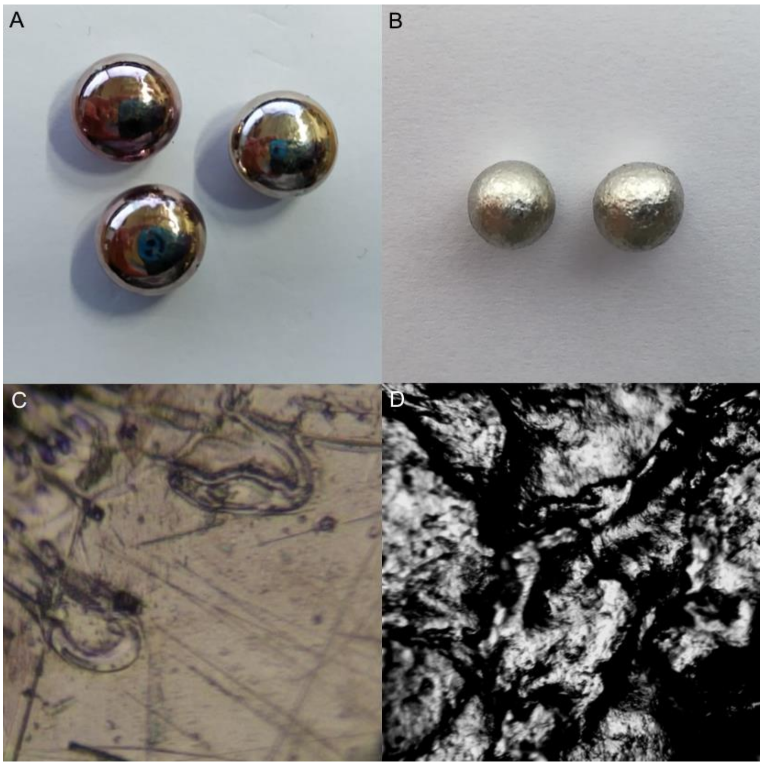

Figure S23: Photos and microscopic images (20x magnification) of Pd milling balls new (A and C) and used (B and D) after more than 140 h of milling. The used Pd milling balls show a surface roughening due to the impacts during milling.

9. Mechanosynthesis of 1,3,5-Triphenylbenzene

For mechanosynthesis of 1,3,5-triphenylbenzene (PPP_{PBM}-15) in a PBM phenylboronic acid (1.258 g, 10.32 mmol, 3 eq.), 1,3,5-tribromobenzene (1.083 g, 3.44 mmol, 1 eq.), palladium black (26 mg, 7.1 mol% of 1,3,5-tribromobenzene) and potassium carbonate (8.510 g, 61.58 mmol) were placed together with twenty-two 10 mm-diameter Si_3N_4 grinding balls in a 45 mL Si_3N_4 grinding jar. The mixture was milled for 4 h at 800 rpm in a Fritsch Pulverisette 7 premium line planetary ball mill. GC-MS analysis of the crude reaction mixture extracted with n-hexane revealed a variety of compounds, namely the product and reaction intermediates (see Table S7) whereas the educt was fully converted.
The same reaction was carried out in MM (PPP_{MM}-6) using phenylboronic acid (732 mg, 6.00 mmol, 3 eq.), 1,3,5-tribromobenzene (630 mg, 2.00 mmol, 1 eq.), potassium carbonate (2.504 g, 18.12 mmol) and two 9 mm Pd milling balls in a PMMA milling jar. The mixture was milled for 8 h in a Retsch MM 400 mixer mill at 30 Hz. Results from GC-MS analysis are shown in Table S7, pointing out that the conversion is much lower than in PBM.
Both reactions PPP_{PBM}-15 and PPP_{MM}-6 are shown as proof of principle. No further product separation or optimization of reaction parameters was performed.

Table S7. GC-MS analyses from synthesis of 1,3,5-triphenylbenzene.

Compound	PPP_{PBM}-15	PPP_{MM}-6
1,3,5-Tribromobenzene	-	86%
3,5-Dibromobiphenyl	25%	Traces
5'-Bromo-m-terphenyl	39%	Traces
1,3,5-Triphenylbenzene	36%	14%

References

[1] D. Gracin, V. Štrukil, T. Friščić, I. Halasz, K. Užarević, *Angew. Chemie - Int. Ed.* **2014**, *53*, 6193–6197.

[2] L. Batzdorf, F. Fischer, M. Wilke, K. J. Wenzel, F. Emmerling, *Angew. Chemie - Int. Ed.* **2015**, *54*, 1799–1802.

[3] I. Halasz, S. A. J. Kimber, P. J. Beldon, A. M. Belenguer, F. Adams, V. Honkimäki, R. C. Nightingale, R. E. Dinnebier, T. Friščić, *Nat. Protoc.* **2013**, *8*, 1718–1729.

[4] K. Užarević, V. Štrukil, C. Mottillo, P. A. Julien, A. Puškarić, T. Friščić, I. Halasz, *Cryst. Growth Des.* **2016**, *16*, 2342–2347.

[5] J. Filik, A. W. Ashton, P. C. Y. Chang, P. A. Chater, S. J. Day, M. Drakopoulos, M. W. Gerring, M. L. Hart, O. V. Magdysyuk, S. Michalik, et al., *J. Appl. Crystallogr.* **2017**, *50*, 959–966.

[6] E. J. Sonneveld, J. W. Visser, IUCr, *J. Appl. Crystallogr.* **1975**, *8*, 1–7.

[7] S. Sasaki, T. Yamamoto, T. Kanbara, A. Morita, T. Yamamoto, *J. Polym. Sci. Part B Polym. Phys.* **1992**, *30*, 293–297.

[8] H. Ohtsuka, Y. Furukawa, M. Tasumi, *Spectrochim. Acta Part A Mol. Spectrosc.* **1993**, *49*, 731–737.

[9] K. Zhang, X.-J. Chen, *AIP Adv.* **2018**, *8*, 025004.

[10] D. A. Fulmer, W. C. Shearouse, S. T. Medonza, J. Mack, *Green Chem.* **2009**, *11*, 1821–1825.

[11] F. Duval, T. van Beek, H. Zuilhof, *Synlett* **2012**, *23*, 1751–1754.

[12] D. J. C. Constable, A. D. Curzons, V. L. Cunningham, *Green Chem.* **2002**, *4*, 521–527.

[13] P. Anastas, N. Eghbali, *Chem. Soc. Rev.* **2010**, *39*, 301–312.

[14] R. S. Sprick, B. Bonillo, R. Clowes, P. Guiglion, N. J. Brownbill, B. J. Slater, F. Blanc, M. A. Zwijnenburg, D. J. Adams, A. I. Cooper, *Angew. Chemie Int. Ed.* **2016**, *55*, 1792–1796.

[15] S. Grätz, B. Wolfrum, L. Borchardt, *Green Chem.* **2017**, *19*, 2973–2979.
